# Exosomes derived from endometriotic stromal cells have enhanced angiogenic effects in vitro

**DOI:** 10.1007/s00441-016-2358-1

**Published:** 2016-02-03

**Authors:** Djana Harp, Adel Driss, Sharifeh Mehrabi, Indrajit Chowdhury, Wei Xu, Dong Liu, Minerva Garcia-Barrio, Robert N. Taylor, Bert Gold, Samantha Jefferson, Neil Sidell, Winston Thompson

**Affiliations:** Department of Obstetrics and Gynecology, Morehouse School of Medicine, 720 Westview Drive, SW, Atlanta, GA 30310 USA; Cardiovascular Research Institute, Morehouse School of Medicine, 720 Westview Drive, SW, Atlanta, GA 30310 USA; Department of Physiology, Morehouse School of Medicine, 720 Westview Drive, SW, Atlanta, GA 30310 USA; Department of Obstetrics and Gynecology, Wake Forest School of Medicine, 1 Medical Center Boulevard, Winston-Salem, NC 27157 USA; Center for Cancer Research, National Cancer Institute, Frederick, MD 21702 USA; Georgia State University, P.O. Box 3965, Atlanta, GA 30302 USA; Department of Gynecology & Obstetrics, Emory University School of Medicine, 1639 Pierce Dr., WMB 4303, Atlanta, GA 30322 USA

**Keywords:** Exosomes, Endometrial stromal cells, Angiogenesis, *MicroRNA*, Infertility

## Abstract

Our objective has been to establish a pro-angiogenic role for exosomes in endometriosis and to determine whether a differential expression profile of cellular and exosomal *microRNAs* (*miRNAs*) exists in endometriosis. We performed an in vitro study of human primary endometrial stromal cells (ESCs) and human umbilical vein endothelial cells (HUVECs). We isolated and characterized exosomes from ESCs from five endometriosis patients and five phase-matched controls. Exosomes were characterized by transmission electron microscopy and NanoSight technology. *MiRNA* was assessed by deep sequencing and reverse transcription with quantitative polymerase chain reaction. Exosome uptake studies were achieved by means of confocal microscopy. The pro-angiogenic experiments were executed by treating HUVECs with ESC-derived exosomes. We observed differential profiles of exosomal *miRNA* expression between exosomes derived from endometriosis lesion cells and diseased eutopic stromal cells compared with exosomes derived from control ESCs. We also demonstrated autocrine cellular uptake of exosomes and paracrine functional angiogenic effects of exosomes on HUVECs. The results of this study support the hypothesis that exosomes derived from ESCs play autocrine/paracrine roles in the development of endometriosis, potentially modulating angiogenesis. The broader clinical implications are that Sampson’s theory of retrograde menstruation possibly encompasses the finding that exosomes work as intercellular communication modulators in endometriosis.

**Electronic supplementary material:**

The online version of this article (doi:10.1007/s00441-016-2358-1) contains supplementary material, which is available to authorized users.

## Introduction

Endometriosis is a benign gynecological disorder characterized by the presence of endometrial glandular tissue and stroma outside of their normal uterine location. Up to 10 % of reproductive-age women in the United States are estimated to have endometriosis, which is associated with debilitating pain, menstrual irregularities, and infertility in as many 30 % of the affected women (Bulun 2009; Verkauf 1987). The precise etiology of endometriosis remains to be elucidated. Commonly accepted theories of the causes of endometriosis include retrograde menstruation, coelomic metaplasia, and lymphatic and vascular dissemination (Macer and Taylor 2012). Patients with endometriosis often have delayed (6–11 years) diagnosis because symptoms are variable and often mimic other gynecological diseases (Fassbender et al. 2015; Leone Roberti Maggiore et al. 2016; Nogales et al. 1993). A definitive diagnosis of endometriosis requires surgery (Gordts et al. 2015). The development of new ways of diagnosis by using exosomal biomarkers might enable earlier diagnosis of the disease (Fassbender et al. 2015).

Angiogenesis, the formation of new blood vessels, has increasingly been recognized as a key pathological factor in endometriosis. Human endometrial tissue is known to be angiogenic throughout the menstrual cycle (Maas et al. 2001). Sampson’s theory of retrograde menstruation posits that endometrial tissue travels retrogradely through the Fallopian tubes and is deposited into the peritoneal cavity, where it establishes a blood supply, proliferates, and forms an implant that is clinically recognized as a lesion (Sampson 1927). Women with endometriosis have been found to have increased retrograde menstruation (Halme et al. 1984). Successful implantation of endometriotic lesions depends on neovascularization, fibrosis, adhesion formation, apoptosis evasion, and neuronal infiltration (Rocha et al. 2013). The exact mechanisms underlying the sprouting and sustainment of endometriosis neovascularization need further clarification. Recent studies, however, demonstrate that inhibitors of angiogenesis block the progression of endometriosis (Edwards et al. 2013; Nap et al. 2004, Wang et al. 2014).

Exosomes have been found to play physiological roles as mediators of intercellular cell signaling between neighboring cells and even amongst distant tissues, and they may act independently but synergistically with soluble growth factors and hormones (Colombo et al. 2014). Exosomes are vesicles ranging from 30 to 150 nm in diameter, are derived from the fusion of multivesicular bodies with the plasma membrane, and are secreted by a variety of living cells; they are composed of a lipid bilayer membrane and contain functionally active proteins, *mRNA* and *microRNA* (*miRNA*) that can be released, rendering them important mediators of intercellular communication (Colombo et al. 2014). The recent discovery of these extracellular organelles containing genetic cargo with a unique signature raises the hope that exosomes will serve as diagnostic markers or therapeutic vehicles (Krause et al. 2015; Momen-Heravi et al. 2015; Tang and Wong 2015).

Among all molecules, the *miRNAs* that are contained within exosomes (Valadi et al. 2007) could affect gene expression and function in target cells, including vascular endothelial cells. Several *miRNAs*, such as *miR*-*21* and *miR*-*126*, have been shown previously to play critical roles in angiogenesis (Wang and Olson 2009). We have chosen to screen these two miRNAs because, in previous literature, they have been widely used by the scientific community as pro-angiogenic indicators in other systems (Bao et al. 2012; Guduric-Fuchs et al. 2012; Xu et al. 2015).

Additionally, endometrial exosomes released into the uterine cavity can transfer information either to the blastocyst or to other cells in the endometrium to influence implantation (Ng et al. 2013). Our hypothesis is that exosomes released from endometriotic stromal cells contribute to the pathogenesis of endometriosis by packaging and delivering specific *miRNAs* in an autocrine and paracrine fashion. In this study, we have examined exosomes as delivery vehicles for pro-angiogenic *miRNA* in endometriosis.

## Materials and methods

### Human subjects and tissue acquisition

The study was approved by the institutional review boards of the Emory University School of Medicine and Morehouse School of Medicine. The primary endometrial stromal cells (ESCs) were obtained from normally cycling, reproductive-age women undergoing surgery for benign gynecological conditions with normal endometrium (NE, *n* = 5) and 5 women with endometriosis who provided eutopic endometrial biopsies (EE, *n* = 5) plus specimens from their endometriosis implants (EI, *n* = 5). For NE controls, endometrial tissues were obtained from patients undergoing surgery for clinical indications, typically infertility, pelvic pain, or suspicion of a pelvic mass, or some combination of findings under protocols approved by the Institutional Review Boards of the Emory University School of Medicine and Morehouse School of Medicine. All the subjects selected were women who had regular menstrual cycles and who had not received hormonal therapy for at least 3 months before surgery (Yu et al. 2014). Absence of endometriosis in the NE group was confirmed after surgical examination of the abdominal and pelvic cavity. Specimens from controls were obtained from normally cycling women undergoing surgery for benign gynecological conditions in which no endometriosis or evidence of endometrial abnormalities was visible. Among the five control subjects, subserosal fibroids were noted in four women, and none were greater than 3 cm in diameter. Endometriosis patients were identified at surgery by expert laparoscopists familiar with the varied appearance of the lesions. Although the subjects were not age-matched, their mean ages were not significantly different (Control, 40.4 ± 5.9; Endometriosis, 34.0 ± 7.8; *P* = 0.53). The secretory menstrual phase according to the day of the reproductive cycle was selected for all biopsies to maximize consistency and was confirmed by histological examination of the endometrial tissues. Written informed consent was obtained prior to surgical removal of endometriotic lesion tissue and endometrial biopsies.

### Cell cultures and reagents

Primary ESCs were prepared from human tissue biopsies according to our published procedure (Ryan et al. 1994). All cultures (passages 3–5) were grown in complete medium to 70–90 % confluence in DMEM/Ham’s F-12 supplemented with 10 % fetal bovine serum (FBS), 1 % non-essential amino acids, 1 % sodium pyruvate, and 1 % penicillin streptomycin and were incubated at 37 °C and 5 % CO_2_. Human umbilical vein endothelial cells (HUVECs) were purchased from Lonza (Walkersville, Md., USA) and were cultured in EBM-2 media supplemented with EGM-2 MV cocktail (Lonza) together with 1 % penicillin and streptomycin.

### Isolation of exosomes

Once the desired confluence was obtained, the culture media were removed, and ESCs were washed twice with 5 ml sterile phosphate-buffered saline (PBS). The ESCs were then cultured for an additional 48 h with growth media containing exosome-depleted FBS. Exosome-depleted FBS was obtained by ultracentrifugation of FBS at 100,000*g* for 16 h at 4 °C. The exosomal fraction from 5 ml culture media was isolated by the Total Exosome Isolation kit (Invitrogen) according to the manufacturer’s recommendations. First, the collected cell culture media were centrifuged at 2000*g* for 30 min at room temperature to remove cells and debris. Second, a half-volume of the exosome isolation solution was added to cell-free culture media, and samples were refrigerated at 4 °C overnight. The mixture was centrifuged at 10,000*g* for 1 h at 4 °C, and the supernatant was removed by aspiration. The pellet was re-suspended in 1× PBS and stored at −80 °C or directly processed for *miRNA* extraction (Li et al. 2015).

### Identification of nanoparticles by nanoparticle tracking analysis

Nanoparticle tracking analysis (NTA) measurements were performed by using a NanoSight NS500 instrument (NanoSight NTA 2.3 Nanoparticle Tracking and Analysis Release Version Build 0025). The size distribution and quantification of exosome preparations were analyzed by measuring the rate of Brownian motion with a NanoSight LM10 system (NanoSight, Wiltshire, United Kingdom) equipped with fast video capture and particle-tracking software. Purified exosomes from NE, EE, and EI samples were diluted in 500 μl of a solution of 1× PBS/5 mM EDTA and disaggregated by using a syringe and needle (29-gauge). After this procedure, the sample was injected into a NanoSight sample cubicle. The mean ± SD size distribution of ESC exosomes was determined, and the mean number of particles per milliliter was compared between endometriosis patients (exosomes derived from EE and EI ESCs) and exosomes derived from eutopic NE cells of healthy subjects (Riches et al. 2014).

### Exosome visualization by transmission electron microscopy

Exosome suspension was loaded into a carbon-coated electron microscopy grid. The sample was fixed with 2.5 % glutaraldehyde in 0.1 M cacodylate buffer for 2 h at 4 °C, followed by a second fixation with 1 % osmium tetroxide in 0.1 M cacodylate buffer for 1 h at 4 °C. After three washes in distilled H_2_0, the sample was stained with 0.5 % aqueous uranyl acetate for 2 h at room temperature. Transmission electron microscopy samples were observed by using a JEOL 1200EX instrument.

### Exosome labeling

Exosomes were first obtained from non-labeled ESCs as described above. The protein content of the exosomes was adjusted to 1.4 μg/ml prior to labeling. Exosomal membrane was labeled with BODIPY TR ceramide, according to the manufacturer’s protocol (Molecular Probes/Invitrogen Life Technologies). Briefly, exosome pellets were re-suspended in 100 μl PBS and stained with 10 μmol/L BODIPY TR ceramide with 594-Alexa-Fluor (red) fluorescence. Excess fluorescent dye was removed by using Exosome Spin Columns (Life Technologies).

### Confocal microscopy and imaging

Cells were cultivated on chamber slides, treated with a final concentration of 28 ng/ml labeled exosomes (red) for 20 min, fixed with 4 % paraformaldehyde at room temperature for 20 min, permeabilized with ice-cold acetone at room temperature for 5 min, and then stained with fluorescent 488-Phalloidin (green) for actin using the manufacturer’s recommendations (Molecular Probes/Invitrogen Life Technologies). Fluorescence images were collected on an inverted microscope (Zeiss LSM-700) via a 40× objective. Zeiss acquisition parameters, including exposure, focus, illumination, and Z stack projection, were controlled by ZEN 2 software (Carl Zeiss Microscopy). For the analysis of the cellular internalization of exosomes, images were also deconvolved by using ZEN 2 software (Chu et al. 2012).

### Endothelial tube formation assay

Sub-confluent HUVECs were harvested, resuspended in medium, and treated with the indicated concentration of exosomes or with PBS as a negative control. The exosome protein concentration was measured by the Coomassie Plus (CP) Protein Assay (Thermo Scientific). Treatment of cells consisted of 50 μg total excreted exosomes extracted from eutopic endometriosis and control endometrial cells. This suspension was seeded (70,000 cells/well) in growth-factor-reduced Geltrex Basement Membrane Matrix (Gibco) on a 96-well plate (BD Bioscience) and incubated up to 24 h at 37 °C with 5 % CO_2_. Tube formation was examined under an inverted microscope and photographed at 40× magnification. Cumulative tube length was measured by using ImageJ software (Schneider et al. 2012). Results are shown as the mean and standard errors of triplicate experiments (Swift et al. 2010).

### Micro-RNA extraction, deep sequencing, and reverse transcription with quantitative polymerase chain reaction

Micro-RNA was extracted from exosomes and cells by using the *mir*Vana *miRNA* isolation kit (Life Technologies) according to the manufacturer’s protocol. *RNA* concentration and relative quality were determined by using a Nanodrop Spectrophotometer (Thermo Scientific). Samples were deemed acceptable if 260/280 ratios were greater than 1.6; 260/230 ratios were greater than 1.0. *miRNA* (1 μg) was reverse-transcribed by using the TaqMan *MicroRNA* Reverse Transcription Kit (Applied Biosystems) following the manufacturer’s protocol. Primers for hsa-miR-21-5p (Cat. no. 4427975-000397), hsa-miR-126-5p (Cat. no. 4427975-000451), and U6 snRNA (Cat. no. 4427975-001973) were used from *miRNA* kits (Applied Biosystems), and real-time quantitative polymerase chain reaction (qPCR) was performed by using the LC480 Lightcycler thermocycler (Roche). *snRNA U6* was used as an endogenous control to normalize expression data. All samples were run in triplicate, and the average value was used to calculate the fold change values. The delta-delta of average Ct/Cp value method was used to calculate the fold change values among samples as previously described (Scarlett et al. 2014). Deep sequencing of total *miRNAs* was carried out by SeqMatic LLC (Fremont, Calif., USA). Illumina sequencing libraries were generated by using the SeqMatic TailorMix *miRNA* sample preparation kit. Sequencing was performed by using the Illumina Genome Analyzer IIx with a 1 × 36 bp single-end read. Data was de-multiplexed by index barcode, and fastq files were generated by using Illumina pipeline (CASAVA). For data quality control, sequence reads were trimmed of the Illumina adapters by using fastx clipper. Reads shorter than 15 bp were removed from subsequent analyses. For data analysis, clean sequence reads were mapped to the human genome (hg19) and to miRBase version 20 by using Bowtie. Parameters were modified to allow for up to one mismatch. Reads mapping to known *miRNAs* were counted. Differential expression and *P*-value estimation were performed by using DESeq for R statistical programming language (Ge et al. 2014).

### Statistics

Real-time qPCR results were expressed as means ± standard deviation (SD) from at least three separate experiments performed in triplicate, unless otherwise stated. Differences between means among the treatment groups were analyzed by using the Student two-tailed *t*-test or one-way analysis of variance (ANOVA) with Holm-Sidak post-tests method where appropriate. Quantitative analysis of branching length in the angiogenesis assay was performed with Pearson correlation analysis. A *P*-value of <0.05 was considered significant. SigmaPlot (version 11.0) and MS Excel software for Windows were used for statistical analysis.

## Results

### Identification of ESC-derived exosomes in culture media

**Fig. 1:**
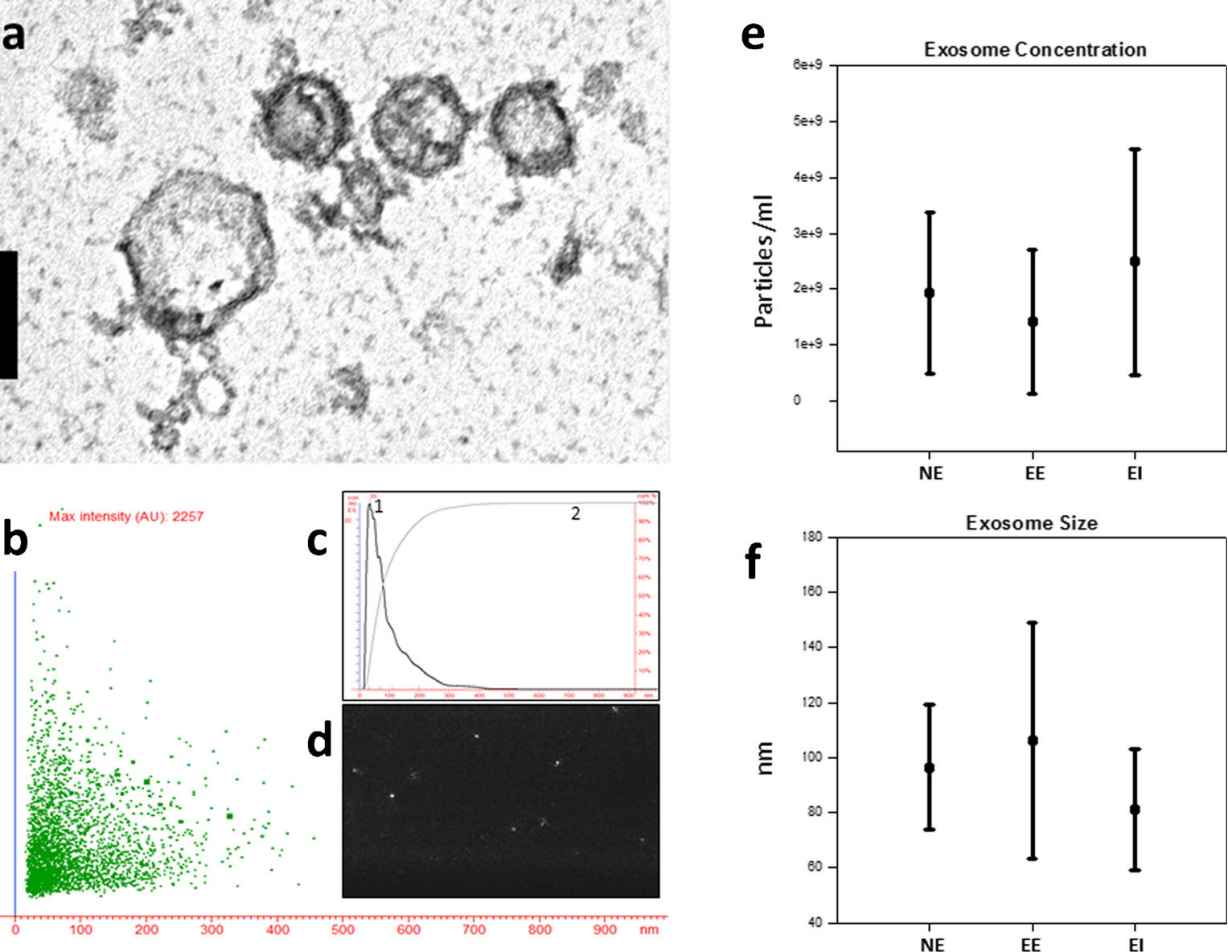
**a** Transmission electron microscopy of exosomes from control endometrial stromal cells (ESCs). *Bar* 100 nm. **b** Scatter plot graph of exosomes by using Nanosight technology shows that the majority of exosomes are between 20 and 200 nm in size with a peak at 35 nm (*X* = particle size, *Z* = count/ml). **c** Curve 1 describes the relationship between particle number distribution (concentration/ml; *left Y axis*) and particle size (in nm; *X axis*); curve 2 describes the correlation between the cumulative percentage distribution of particles (percentile; *right Y axis*) and particle size (*X axis*). **d** Still figure of exosome scatter nanoparticle tracking in a NanoSight video (Supplementary video 1). **e** Repeated-measure analysis of variance showing scatter plot column comparison of means of exosome concentrations between the three groups (*NE* normal endometrium, *EE* eutopic endometrium, *EI* endometriosis implants). No measurable differences between the three groups were observed (*P* = 0.655). **f** Scatter plot column comparison of means of exosome sizes between the three groups. No measurable differences between the three groups were observed (*P* = 0.529). *Error bars* are the standard deviations of the different readings from different isolations from T-75 flasks after 24 h of changing to exosome-free medium

The ESC exosome purification procedure was validated by using electron microscopy and NanoSight analysis. Figure 1a shows an example of a transmission electron micrograph of a representative exosome preparation. Further characterization of exosomes, including size measurement and quantification, from healthy (Fig. 1b–d, Supplementary video 1) and endometriosis subjects was performed by using NanoSight analysis, which demonstrated the purity of the vesicles, with a peak size at 35 nm. This confirmed that the ESCs of control and endometriosis subjects released exosomal vesicles into their conditioned medium. No measurable differences were observed in size or yield (Fig. 1e, f).

### Autocrine internalization of exosomes by ESCs

**Fig. 2:**
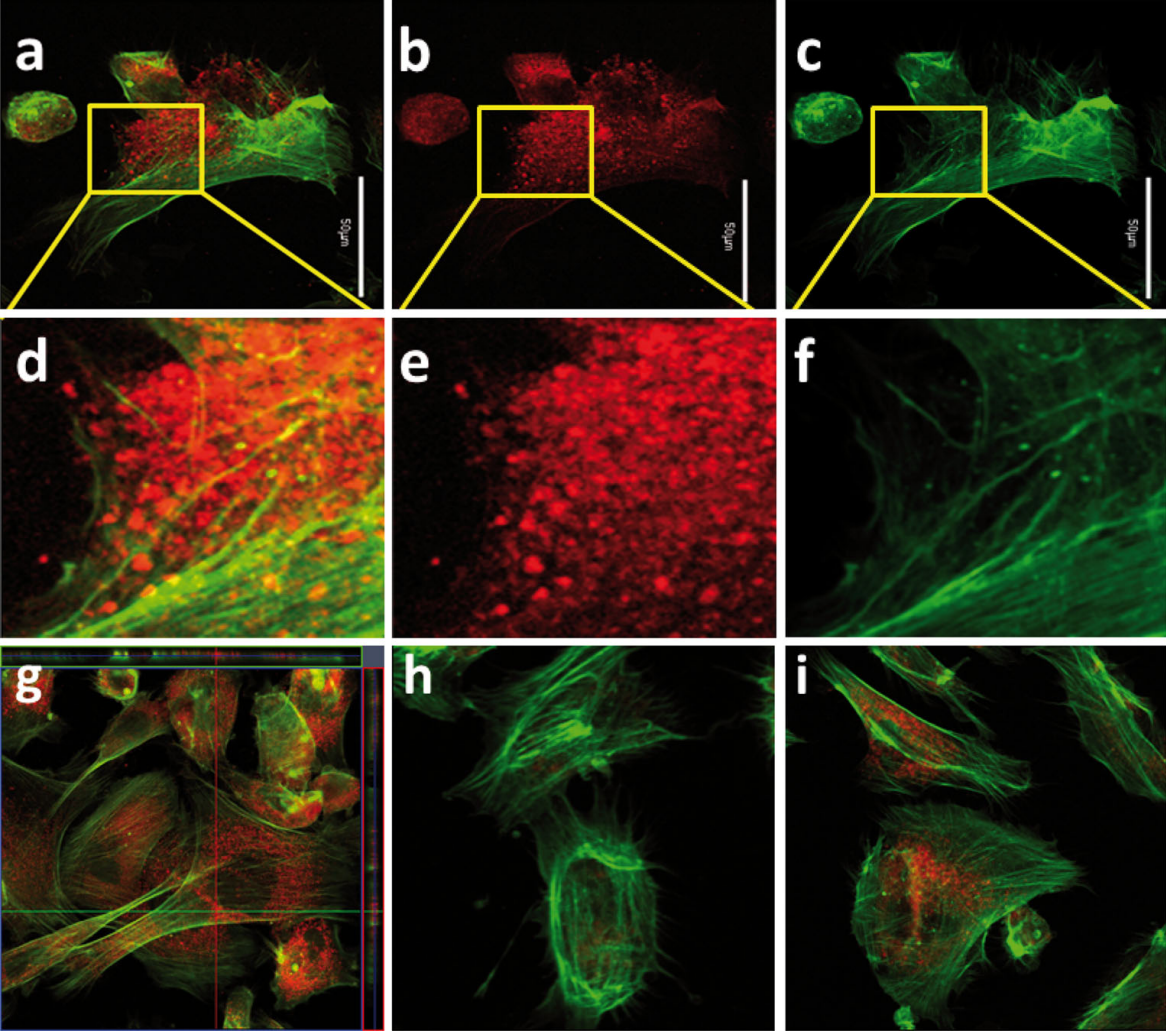
Uptake of exosomes by NE ESCs. The protein equivalent of 28 ng/ml labeled exosomes (*red*) was incubated for 20 min with ESCs, which were then fixed in 4 % paraformaldehyde at room temperature for 20 min, subsequently permeabilized with ice-cold acetone at room temperature for 5 min, and finally labeled with 488-Phalloidin (*green*). Similar results were obtained when we used NE and EE exosomes, and when we used EE cells. **a** ESCs labeled with 488-Phalloidin (*green*). **b** Exosomes labeled with 594-Alexa-Fluor (*red*). **c** Merged image. **a–c** Magnification: 40×. **d–f** Higher magnification of *boxed areas* in **a–c**. **g** Z-stack slices running apical to basolateral in ESCs showing the ZX (*upper box*) and ZY (*right side box*) cross sections of the selected cells (Supplementary video 2) showing that the exosomes (*red*) are located inside the ESCs and not on the top or bottom. **h** Uptake by the ESCs of exosomes after 2 min of treatment. **i** Uptake by the ESCs of exosomes after 8 min of treatment

We hypothesized that exosomes work in an autocrine/paracrine fashion and can be internalized by nearby cells to affect function in the uterine microenvironment or peritoneal cavity. The internalization of labeled purified exosomes was visualized by confocal fluorescence microscopy in ESCs (Fig. 2a–g, Supplementary Fig. S1, Supplementary video 2). We observed that the internalization of exosomes occurred within 2 min of exposure to treatment, suggesting a fast uptake mechanism (Fig. 2h, i). In these experiments (Fig. 2), we observed the uptake of exogenous purified NE exosomes by NE ESCs. Similar results were found when we used EE exosomes on NE cells, and when we used EE cells. The findings support the ability of exosomes to influence cell behavior via autocrine and/or paracrine routes.

### Pro-angiogenic effects of exosomes derived from ESCs

**Fig. 3:**
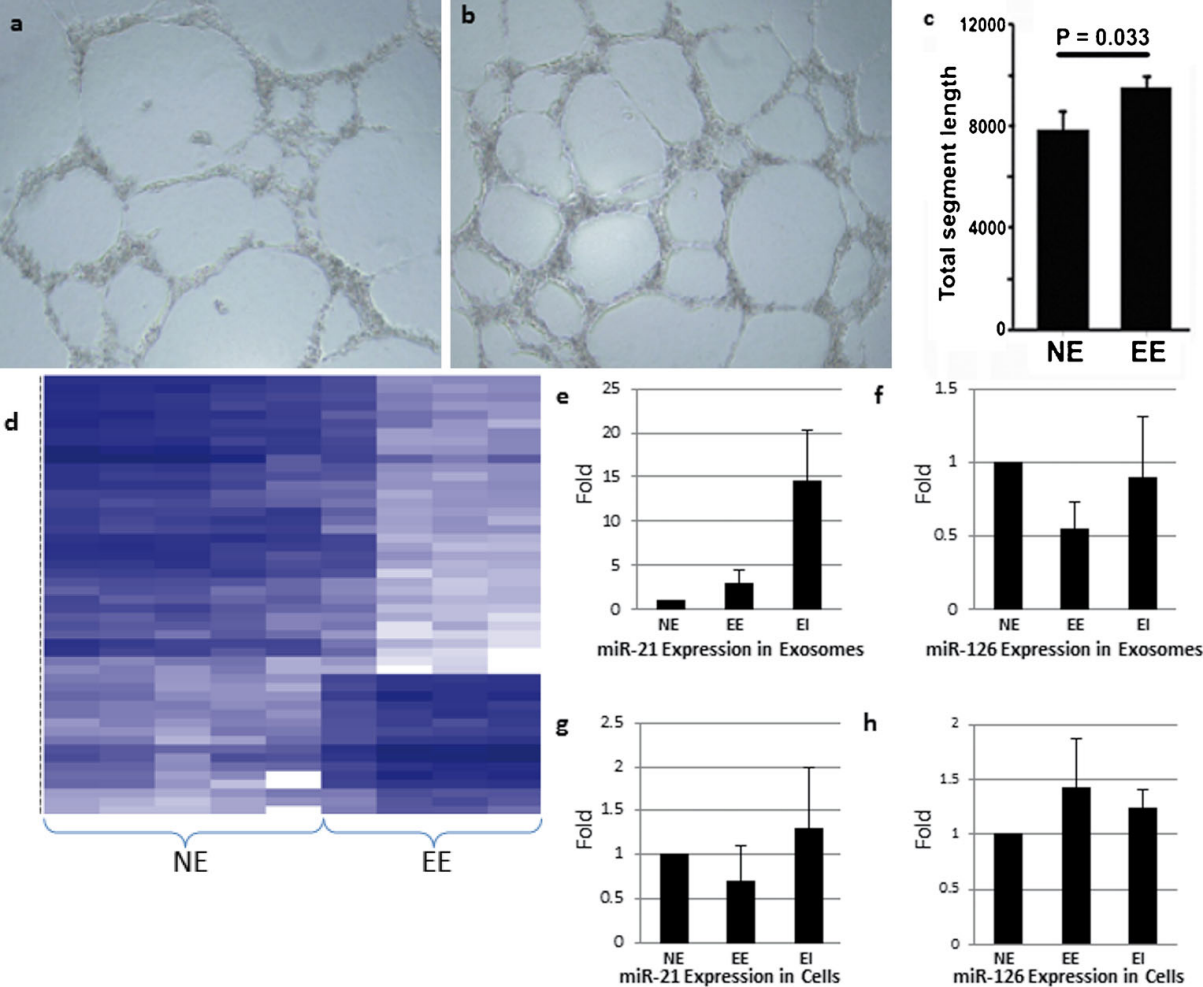
**a** HUVECs treated with exosomes extracted from control cells at 24 h. **b** HUVECs treated with exosomes extracted from endometriosis patient cells at 24 h. **c** Significant increase of total segment branch length is noted on HUVECs treated with exosomes extracted from EE cells compared to those from NE cells (*error bar* SD). **d** Heat-map from deep-sequencing depicting the most significant differential expression of *miRNAs* extracted from exosomes of NE and from EE. **e**, **f** *miR*-*21* and *miR*-*126* expression, respectively, revealed by reverse transcription and quantitative polymerase chain reaction (RT-qPCR) of RNA extracted from exosomes from NE, EE, and EI samples. EI cells showed an 11-fold increase in *miR*-*21* expression versus the healthy control eutopic ESC exosomes (*P* < 0.0001). No significant difference in *miR*-*126* expression was found among samples. **g**, **h** *miR*-*21* and *miR*-*126* expression, respectively, revealed by RT-qPCR of RNA extracted from cells of NE, EE, and EI samples. No significant differences between groups were shown between intracellular expression levels of *miR*-*21* and *miR*-*126* extracted from the producing cells in culture

HUVECs plated on Geltrex Basement Membrane Matrix in medium treated with exosomes extracted from eutopic endometriosis ESC medium had greater tube formation compared with those treated with exosomes extracted from control ESC medium (Fig. 3a, b). A significant increase (*P* = 0.033) of total segment branch length was noted on HUVECs treated with exosomes extracted from endometriosis cells compared with control cells (Fig. 3c).

### Differential expression of exosomal pro-angiogenic *miRNAs*

Deep sequencing data also showed that several *miRNAs* extracted from exosomes of NE and EE were differentially expressed between the two groups (Fig. 3d). The deep sequencing results led to the identification of several *miRNAs* that have yet to be characterized and confirmed in a larger patient sample size. As angiogenesis has been recognized to be a key pathological factor in endometriosis, we found, from our deep sequencing heat-map data, that some *miRNAs* could be predicted to be involved in the angiogenesis process. Since our endothelial tube assay showed pro-angiogenic effects in EE-treated HUVECs, we analyzed two known pro-angiogenic *miRNAs* (*miR*-*21* and *miR*-*126*) to determine whether differential expression occurred. We investigated the expression in total *miRNA* extracted from exosomes secreted by NE, EE, and EI ESCs. Whereas the data did not reveal significant differences in *miR*-*126* among samples, EI cells showed an 11-fold increase in *miR*-*21* expression versus the healthy control eutopic ESC exosomes (*P* < 0.0001; Fig. 3e, f). Intracellular expression levels of *miR*-*21* and *miR*-*126* extracted from the producing cells in culture showed no significant differences between groups (Fig. 3g, h).

## Discussion

Angiogenesis is necessary for the establishment and proliferation of endometriotic lesions (Shifren et al. 1996). Many molecules and various mechanisms involved in lesion angiogenesis are currently under study (Araldi et al. 2015; Kazanis et al. 2015; Li et al. 2015).

 We have demonstrated exosome release from ESCs and confirmed the presence of exosomes by morphological analysis and dimensions. We have shown that the exosomes are rapidly uptaken by ESCs in vitro (Fig. 2). No morphological difference has been noted following the uptake of exosomes from NE cells and from EE cells by either cell type. We postulate that specific surface ligands allow the highly efficient and targeted uptake of these vesicles by recipient cells, as has been shown in other cell types (Schneider and Simons 2013). We hypothesize that ESC-released exosomes are taken up by and have a pro-angiogenic effect on endothelial cells that helps to promote tube formation. We have further shown differential exosomal angiogenic activity on HUVECs in the in vitro Matrigel assay. Endothelial cells are integrally necessary for the formation of new blood vessels. We have observed increased branching when the HUVECs are treated with EE exosomes compared with NE exosomes (Fig. 3a–c). These data suggest that exosomes play a role in promoting endothelial cell formation and angiogenesis in endometriosis. Exosomes from a variety of other cell types have been shown to exert a pro-angiogenic effect on endothelial cells and on stromal cells (Mineo et al. 2012; Paggetti et al. 2015; Pascucci et al. 2014). From our data, we propose that exosomes can play an important mediator role in the angiogenesis process in vitro in a paracrine fashion.

In the present study, we show that ESCs release biologically functional exosomes that can facilitate angiogenesis in a validated in vitro assay. Exosomes carry a variety of bioactive molecules: *RNAs*, *DNAs*, proteins, and lipids (Colombo et al. 2014). The various types of response in target cells induced by exosomes derived from parent cells are probably determined by their *miRNA* content (Pascucci et al. 2014; Shabbir et al. 2015). Our deep sequencing data suggest that endometriosis patients have a differential expression of exosomal *miRNA* patterns compared with controls (Fig. 3d). This signifies that differential pathogenic roles and altered mechanisms are involved in exosomal intercellular communication in the peritoneal and intrauterine milieu.

Our results indicate that at least one of these factors, namely the pro-angiogenic *miRNA*, *miR*-*21*, is differentially expressed in lesion exosomes of endometriosis-derived samples (EI) relative to normal (NE) exosomes. Transferred exosomal *miRNA* can reprogram the recipient cells. The linkage between exosomal *miRNAs* and their effects in the recipient cells remains to be fully elucidated. However, evidence to date indicates that exosomes are powerful mediators of intercellular communication (Tang and Wong 2015). We propose that the exposure of endothelial cells to ESC exosomes leads to angiogenesis, with some of the effects possibly being attributable to the overexpression of *miR*-*21*. Exosomes containing *miR*-*21* have been previously reported to stimulate cell angiogenesis in other systems (Bao et al. 2012; Guduric-Fuchs et al. 2012; Xu et al. 2015). We speculate that exosome-derived *miRNAs* are potential angiogenic targets that are relevant to endometriosis sequelae such as abnormal implantation and infertility. Thus, these exosome-specific *miRNAs* would be present in the uterine and peritoneal microenvironments mediating cross-talk essential for the constellation of secondary effects of endometriosis. The work presented here sets the basis for a path toward the discovery of novel effective biomarkers for early diagnostics and potential new drug targets.

**Fig. 4:**
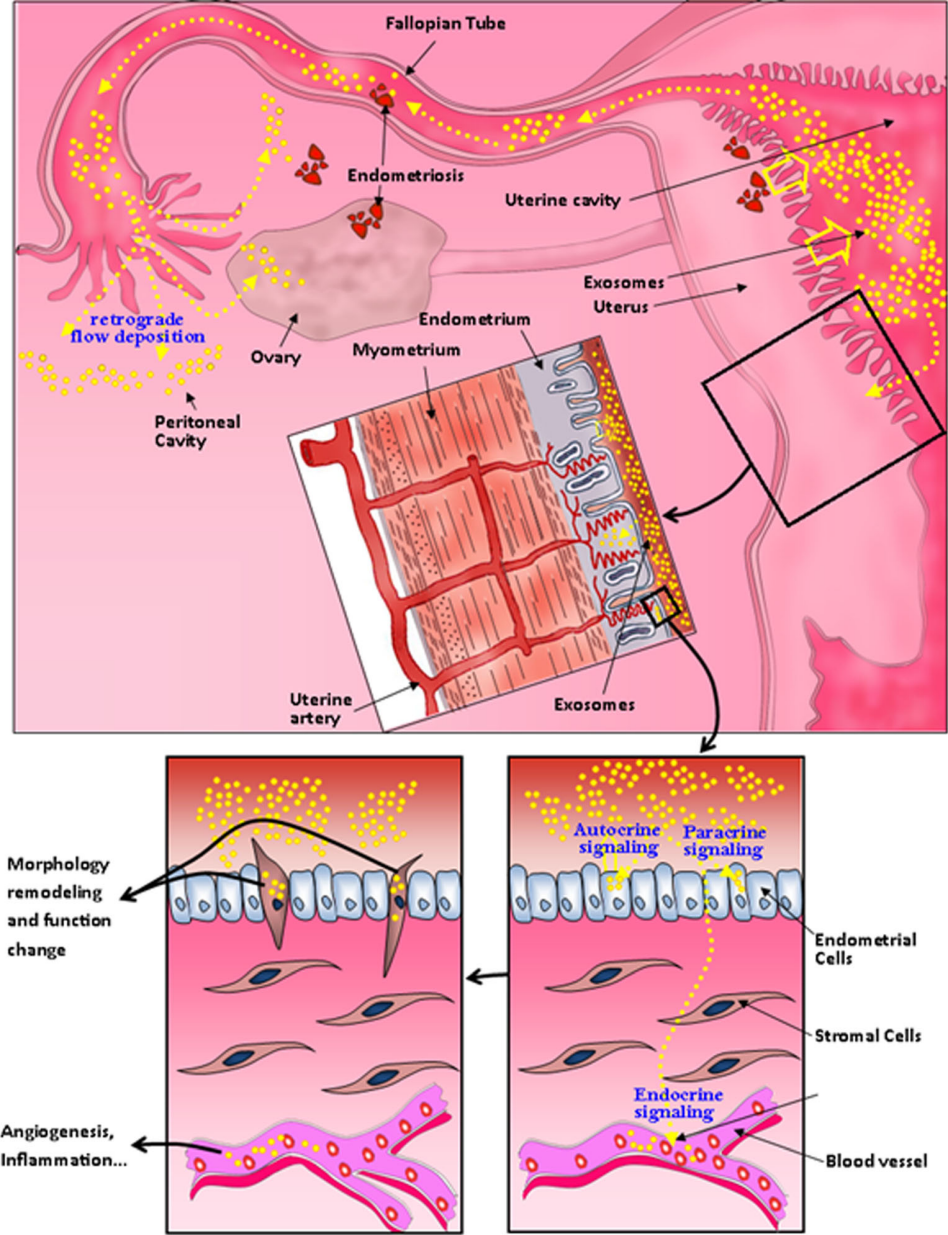
Suggested model depicting exosome shedding in the endometrial cavity working in an autocrine, paracrine, and endocrine manner. Exosomes may also be shed in a retrograde fashion with menstrual flow and be taken up by cells within the peritoneal cavity or shed by stromal cells in the menstrual flow

This study supports the hypothesis that exosomes derived from endometriotic stromal cells play autocrine/paracrine roles in the endometrial and peritoneal microenvironments, modulating angiogenesis. Figure 4 shows our suggested model of action of exosomes in the uterine microenvironment. Additionally, exosomes can migrate further into other tissues through blood vessels (endocrine; De Toro et al. 2015; Ng et al. 2013; Salomon et al. 2014; Simpson et al. 2008) and be transported to the peritoneal cavity through a retrograde flow deposition system. Furthermore, these lesion exosomes might modify angiogenesis in the local peritoneal environment.

The broader clinical implications discussed are built on Sampson’s theory of retrograde menstruation, a theory that supports the idea that exosomes work as intercellular communication modulators in endometriosis. We suggest a model in which exosomes secreted from diseased endometrial cells stimulate the pro-angiogenic propensity of nascent lesions in the peritoneum to adopt a new vasculature and cause inflammatory changes that lead to pelvic pain and infertility. This pathogenic intercellular transfer of genetic material mediated by exosome carriers could pave the road to new diagnostic and therapeutic tools in the field of reproductive biology.

## Electronic supplementary material

Below is the link to the electronic supplementary material.

Supplementary Fig. S1 Z-stack slices from the apical (*top left*) to basolateral (*bottom right*) region of the cells. The distance between each Z-cut image is 0.43 μm (20 pictures in total). This shows that the exosomes (*red*) are located inside the cells and not on the top or bottom (Supplementary video 2). (GIF 283 kb)

High Resolution Image (TIF 470 kb)

Supplementary video 1 Video of scatter exosome tracking by using NanoSight technology. Nanoparticle Tracking Analysis (NTA) utilizes the properties of both light scattering and Brownian motion in order to obtain the particle size distribution of samples in liquid suspension. A laser beam is passed through the sample chamber and the particles in suspension in the path of the beam scatter light in such a manner that they can be easily visualized at 20× magnification via a long-working-distance microscope onto which is mounted a video camera. The camera captures a video file of the particles moving under Brownian motion. The NTA software tracks many particles individually and, by using the Stokes Einstein equation, calculates their hydrodynamic diameters. The NanoSight system provides high resolution particle size, concentration, and aggregation measurements. (MP4 5690 kb)

Supplementary video 2 Video of the Z-stack slices from the apical to basolateral region of the cells. The distance between the apical to basolateral region of the cells is 0.43 μm (20 frames in total, Supplementary Fig. S1). This shows that the exosomes (*red*) are located inside the cells and not on the top or bottom. (AVI 15360 kb)
