# Supplementary figures and images for: Exosomes derived from endometriotic stromal cells have enhanced angiogenic effects in vitro
Source: Cell Tissue Res. 2016 Feb 3;365:187–96. doi: 10.1007/s00441-016-2358-1 (PMC4917586; doi:10.1007/s00441-016-2358-1)

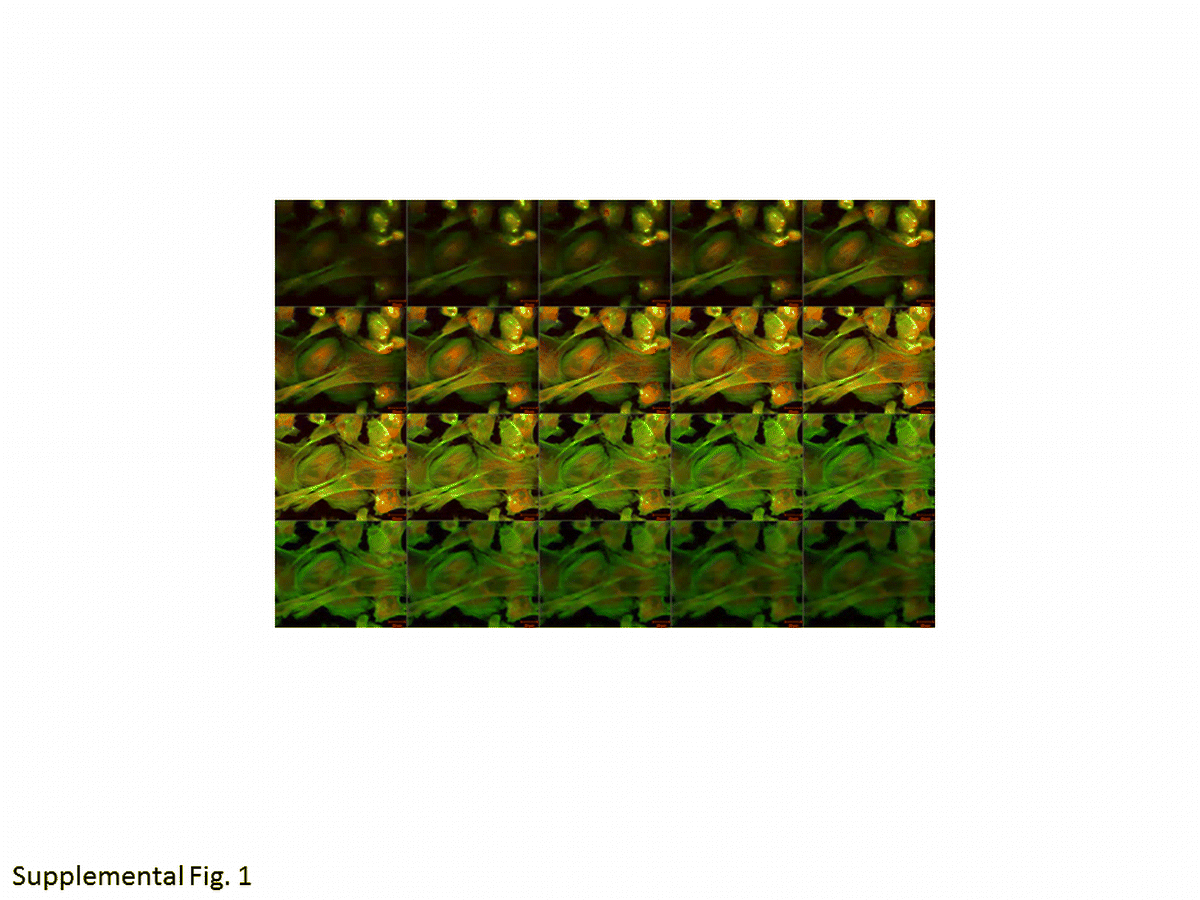

Supplement: Supplementary file 1 — Z-stack slices from the apical (top left) to basolateral (bottom right) region of the cells. The distance between each Z-cut image is 0.43 μm (20 pictures in total). This shows that the exosomes (red) are located inside the cells and not on the top or bottom (Supplementary video 2). (GIF 283 kb) [file 441_2016_2358_Fig5_ESM.gif]

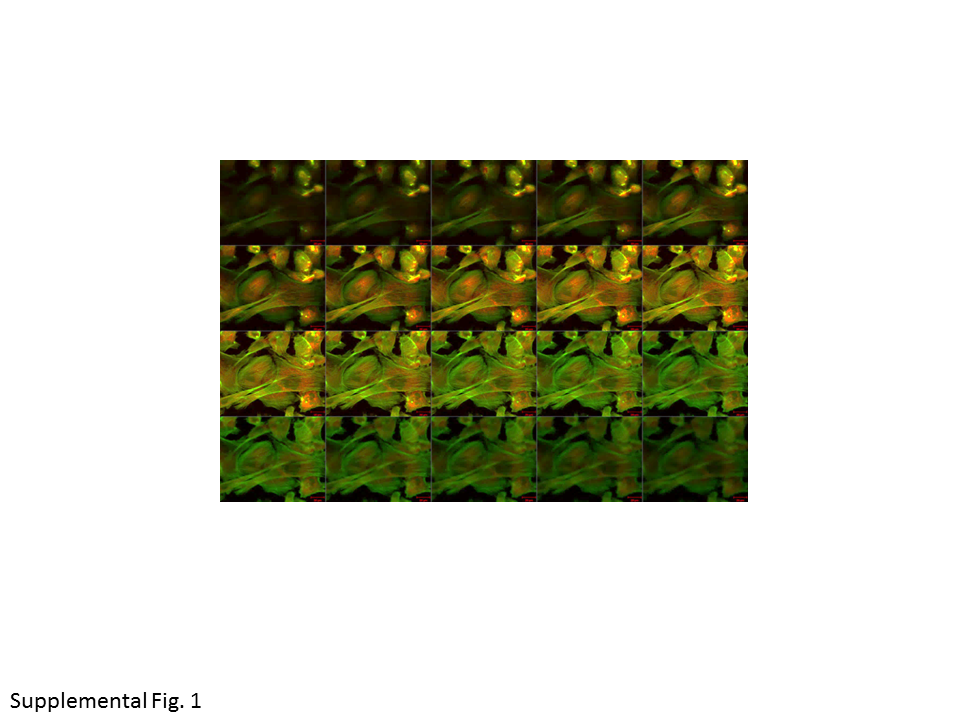

Supplement: Supplementary file 2 — High Resolution Image (TIF 470 kb) [file 441_2016_2358_MOESM1_ESM.tif]
